# The need for reporting negative results - a 90 year update

**Published:** 2017-07-31

**Authors:** Brian D. Earp

**Affiliations:** Departments of Psychology and Philosophy, Yale University, New Haven, Connecticut, United States Email: brian.earp@gmail.com

In January of 1927, Dr. Richard D. Mudd of Detroit pub-lished a letter in the Journal of the American Medical Associa-tion, seeking to vindicate his grandfather, Dr. Samuel A. Mudd, against charges of conspiring in a murder [1]. The victim was U.S. President Abraham Lincoln; the murderer, actor John Wilkes Booth (see Appendix). In this editorial, I, an erstwhile actor, would like to vindicate my own grandfather, Dr. John Rosslyn Earp, for a letter he published on the same day, just one column over, in the very same issue of the journal [2]. But I mean “vindicate” in its other sense—to prove correct—as we shall see.

**Figure 1.**
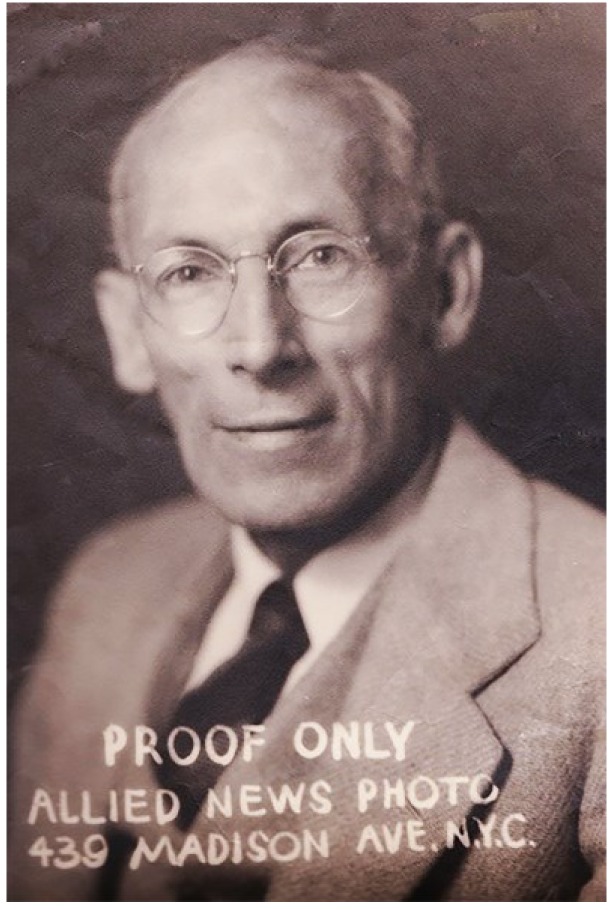
Photograph of John Rosslyn Earp, taken circa 1930

I never knew my grandfather. He died in 1941 at the age of 49, more than four decades before I was born. My father, his son, hardly knew him either: he was only 7 when “Ros” passed away from longstanding health problems, leaving him and his siblings to the care of their mother. I had been told that Grandpa Earp—no relation to Wyatt—was at one point the Director of Public Health for the State of New Mexico [3]. I knew that he'd emigrated from somewhere in England around the turn of the last century. That, and an impression I had from an old photographic proof balanced atop a bookcase in my childhood home, was about it (Figure 1).

In 2013, I took a break from my acting career to study the history and philosophy of science at the University of Cam- bridge. My preoccupation at the time, which has not abated, was the public and professional “crisis of confidence” affect¬ing among other fields medicine and social psychology [4-6]. The term “crisis of confidence” refers to the “unprecedented level of doubt” experienced by many contemporary scientists about the reliability of reported findings in the literature [7].

Why all the doubt? There are several reasons. Anonymous surveys of practicing scientists have shown widespread use of “questionable research practices,” including “p-hacking,” selective reporting of measures or outcomes, and HARKing —hypothesizing after the results are known—all of which in-crease the likelihood of generating Type 1 errors [8-11]. Moreover, critiques have been raised about the reward struc¬ture of science which favors non-stop “productivity” and head¬line-grabbing conclusions over painstaking methodology [12-15]. And a series of high-profile apparent failures to repli¬cate major findings from prior studies has sent shockwaves through the scientific community [16,17].

All of this has combined to create a sense of genuine worry: how much of what we think we know do we actually know? Controversially, at least one prominent meta-scientist, John Ioannidis, has estimated that “most published research findings are false” [18].

The hardest-hit field seems to be psychology (which to its credit has also taken up the vanguard for reform) [19,20], with biomedicine and related disciplines trailing not so far behind [21-23]. Since I had studied the former subject as an under-graduate student, I was familiar with an eerily similar crisis in that field from the 1970s, as a result of which leading practi-tioners sought to root out problems in the way they conducted, evaluated, and published their empirical research [24]. One of the biggest problems to get spotlight treatment was the failure of most journals to publish “negative” results.

In a now-famous article published in 1975, Professor An-thony Greenwald, then of Ohio State University, discussed what he called the “Consequences of prejudice against the null hypothesis” [25]. As he wrote, the lack of a dependable “home” for negative findings creates “a dysfunctional re-search-publication system.” Not only are there “relatively few publications on problems for which the null hypothesis is (at least to a reasonable approximation) true,” but, even among those, “a high proportion will erroneously reject the null hy-pothesis.”

In short, Greenwald identified what is now termed “publica-tion bias” in favor of “statistically significant” findings—a bias that has featured prominently in contemporary discussions about the potential causes of the so-called “replication crisis”[26-28].

The idea is simple. If 20 labs, say, run essentially the same experiment, and only one of them gets it to “work,” chances are good that the apparent finding from this one “lucky” lab is actually a statistical fluke. But since journals—and especially high-impact journals—have had a historical tendency to pub-lish only positive findings, it is this probably-a-fluke result that will end up enshrined in the scientific record [29].

The “negative” results, by contrast, from the 19 other labs in our dummy example—or perhaps the 19 previous versions of the same study from the original lab, recast as “pilot” experi-ments when they didn't pan out—won't typically be written up and submitted, much less published in a prominent journal. Instead, they get “filed away” in the researcher's bottom drawer (the so-called “file drawer” problem), never to be seen again [30,31].

The literature, then, gets skewed in the direction of impres-sive-looking errors, which, for obvious reasons, can't be repli-cated later on. In a clinical context, this “skew” may have se-rious ethical implications for the protection of patient health and well-being. As the editor-in-chief of this journal notes, “selective publication [of] trials can skew the apparent risk-benefit ratio of the drug towards the latter and generate an unrealistic bias, thereby potentially slanting the accuracy of evidence-based medicine” [32].

Needless to say, medical treatments need to be based on accurate research. Basing them on something else is not only unethical (because of the unjustified risk it poses to patients and study participants); it is also an extraordinary waste of resources [33]. Selectively publishing “positive” findings makes these problems worse.

So what can be done? In the course of researching this issue, I stumbled across a paper with a pertinent title that I thought might offer a solution: “The Need for Reporting Negative Re-sults.” The source? Journal of the American Medical Associa-tion—volume 88, number 2. The year? 1927. The author? J. R. Earp, my grandfather [2].

I had no idea he had ever written on the subject (to speak of chills and spines is to get it right). What follows then is his prophetic letter in full, with a few minor edits for ease of reading:

*To the Editor:—One of the things we practitioners sometimes neglect is the reporting of failures. In THE JOURNAL, Oct. 2, 1926, Dr. Richard L. Sutton, with proper scientific reserve, reported the treatment of six consecutive cases of warts with intramuscular injections of sulpharsphenamine. As a result of this communication,* *I venture to guess that not less than a hundred physicians, perhaps several hundred, injected sulpharsphenamine into patients with warts. Supposing that 99 per cent get negative results, what happens? Each of them gives up the method as a failure and does not say anything more about it, and the treatment remains on record as an un¬disputed success. Possibly 1 per cent who meet with suc¬cess will communicate with Dr. Sutton, so that by and by he will have quite an impressive series of cases, compa¬rable with the mercurochrome successes published in a recent number of THE JOURNAL. ...*

To practice what I am preaching, let me now report that on November 30, I injected 0.4 g of sulpharsphena- mine [into] the left buttock of E. M. B., a girl, aged 18, who was at that date complaining of the presence of twenty-four warts distributed mostly over the hands and arms. At the present date, there are twenty-eight warts, and evidence of regressive changes in the original twen¬ty-four has not been seen.

The problem is plain to see; the “need for reporting negative results” is equally apparent [34]. But one-off letters to the edi-tor by conscientious doctors like my grandfather will not suf¬fice to address the root of the problem. What is needed is top-down leadership from journals themselves: not only pas¬sively allowing for the submission of negative findings, but actively welcoming them and even seeking them out. In fact, it should be no harder to publish a high-quality study with “null” results—including unsuccessful attempts at replication—than a high-quality study that purports to show an effect.

There are some signs of progress. Articles with “replication” in the title are now being published on a regular basis [35-42]; there is even a dedicated Journal of Articles in Support of the Null Hypothesis (although it is not especially well-known). But there is still a lot of room for improvement. In a recent review of 1151 journals, researchers found that only 3% ex-plicitly stated that they accepted replications; 63% did not state as much but also did not discourage them; 33% discouraged them implicitly by stressing novelty in solicited submissions; and 1% actively frowned on replications by stating that they did not publish them [43].

Against this backdrop, where does the Journal of Clinical and Translational Research (JCTR) stand? In the founding editorial for this journal, the editor states that JCTR encour¬ages the publication of negative results for two main reasons in addition to counteracting the “skewing” problem already men-tioned [32]:

(1)*publication of negative data, especially when ob¬tained in a technically sound study ... provides cuesas to why a certain procedure or process did not work and steers research efforts away from failure. In that* *sense, something not working can be considered ‘part'* *of the mechanism.*

(2)*negative results prevent colleagues from conduct¬ing redundant work, saving animals and valuable resources in the process. An expedient trajectory to the clinical setting, during which redundancy is mini¬mized, is ultimately beneficial for everyone involved in translational and clinical research as well as the target group (i.e., patients).*

It is with these points in mind that I am happy to introduce, on behalf of my co-editors Emma Bruns and Michal Heger— as well as the entire journal staff—this special issue dedicated entirely to the publication of negative results. Though I never had a chance to meet him, something tells me Grandpa would be proud.

## Figures and Tables

**Figure:**
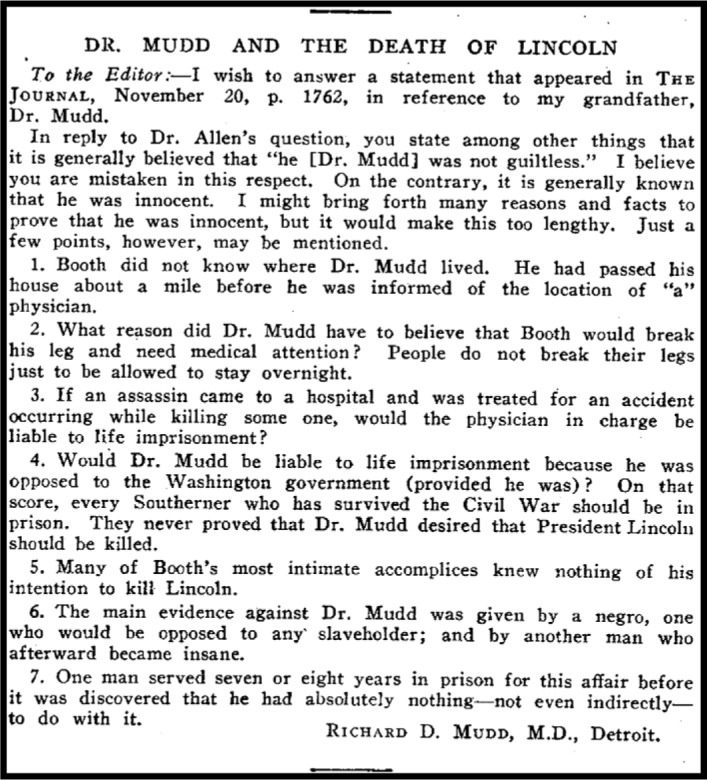
Letter from Dr. Mudd. JAMA. 1927;88:119.
